# Evaluation of an Artificial Intelligence System for the Detection of Diabetic Retinopathy in Chinese Community Healthcare Centers

**DOI:** 10.3389/fmed.2022.883462

**Published:** 2022-04-11

**Authors:** Xiuqing Dong, Shaolin Du, Wenkai Zheng, Chusheng Cai, Huaxiu Liu, Jiangfeng Zou

**Affiliations:** Department of Ophthalmology, Dongguan Tungwah Hospital, Dongguan, China

**Keywords:** artificial intelligence, diabetic retinopathy, community healthcare, color fundus photography, sensitivity, specificity

## Abstract

**Objective:**

To evaluate the sensitivity and specificity of a Comprehensive Artificial Intelligence Retinal Expert (CARE) system for detecting diabetic retinopathy (DR) in a Chinese community population.

**Methods:**

This was a cross-sectional, diagnostic study. Participants with a previous diagnosis of diabetes from three Chinese community healthcare centers were enrolled in the study. Single-field color fundus photography was obtained and analyzed by the AI system and two ophthalmologists. Primary outcome measures included the sensitivity, specificity, positive predictive value, and negative predictive value with their 95% confidence intervals (CIs) of the AI system in detecting DR and diabetic macular edema (DME).

**Results:**

In this study, 443 subjects (848 eyes) were enrolled, and 283 (63.88%) were men. The mean age was 52.09 (11.51) years (range 18–82 years); 266 eyes were diagnosed with any DR, 233 with more-than-mild diabetic retinopathy (mtmDR), 112 with vision-threatening diabetic retinopathy (vtDR), and 57 with DME. The image ability of the AI system was as high as 99.06%, whereas its sensitivity and specificity varied significantly in detecting DR with different severities. The sensitivity/specificity to detect any DR was 75.19% (95%CI 69.47–80.17)/93.99% (95%CI 91.65–95.71), mtmDR 78.97% (95%CI 73.06–83.90)/92.52% (95%CI 90.07–94.41), vtDR 33.93% (95%CI 25.41–43.56)/97.69% (95%CI 96.25–98.61), and DME 47.37% (95%CI 34.18–60.91)/93.99% (95%CI 91.65–95.71).

**Conclusions:**

This multicenter cross-sectional diagnostic study noted the safety and reliability of the CARE system for DR (especially mtmDR) detection in Chinese community healthcare centers. The system may effectively solve the dilemma faced by Chinese community healthcare centers: due to the lack of ophthalmic expertise of primary physicians, DR diagnosis and referral are not timely.

## Introduction

The diabetic population will be 552 million around the world by 2030, based on a previous article (1). Diabetic retinopathy (DR) is one of the most common microvascular complication of diabetes, which reduces quality of life (2, 3). In the physical course of diabetes, ~35% of patients will have fundus abnormalities, and 10% of patients will develop into severe vision-threatening DR (4). In China, the prevalence of DR in diabetes was 14.9 and 2.5% in the prediabetic population due to some study (5, 6). Although the variation in the population and study methodology may affect the prevalence estimates, it is still a large number of patients at risk of DR, since the estimated number of patients with diabetes in the mainland is 129.8 million (7). DR is a major public health problem in China and is noteworthy.

DR often leads to vision loss and has become the principal cause of blindness in working-age populations worldwide (8). There is widespread knowledge that the detection of DR is important, and prompt and proper treatment of early DR can effectively prevent visual impairment (9). However, limited by the different economic and medical levels in each province or city, DR screening remains patchy in China. Furthermore, only a well-trained ophthalmologist can complete the assessment of fundus photographs. Doctors without the professional training in ophthalmology may not offer an accurate diagnosis and prompt treatment, who are the major participants in DR screening in China. Along with the rapid development of deep learning and deep neural networks, artificial intelligence (AI) programs have been applied to identify different photographs of many diseases, including DR, and the system has shown good sensitivity and specificity (10, 11). The popularization of AI systems for DR screening can reduce the workload of ophthalmologists and the demand for doctors.

IDx-DR (Digital Diagnostics, Coralville, USA), Selena (EyRIS, Singapore), Google Inception, and Robert Bosch GmbH (Gerlingen-Schillerhöhe, Germany) are the most widely used systems (12). IDx-DR was the first artificial intelligence medical device approved by the American Food and Drug Administration. In a pilot study, for a population of 819 patients, the system achieved 87.2% sensitivity and 90.7% specificity. Furthermore, the recognition rate of IDx-DR in macular edema is gratifying, which is about 84%. Moreover, these results can only be obtained from fundus photographs (13). Selena is another well-known AI system that is used for disease detection. The model was developed by the Singapore Eye Research Institute. The sensitivity was 90.5% and the specificity was 91.6% in the identification of DR, which matched the results obtained by the human evaluators (14). Although the reliability of detecting and grading DR has been reported by many studies, AI systems have not been widely used in DR screening in community populations. AI system training datasets from tertiary hospitals and large medical centers usually have older age, longer duration of diabetes and higher prevalence of complications and comorbidities (15). In contrast, community-based diabetics are generally in stable condition and have fewer complications, and a lower prevalence of diabetic retinopathy. The application value of AI is to relieve the workload of ophthalmologists and improve the efficiency of screening. Therefore, its application scenario in the real world should be community healthcare centers, rather than in large general hospitals or ophthalmic centers. Because it is the community healthcare center that have a limited number of ophthalmologists and a large population of diabetics who have never been screened for DR. Thus, it is necessary to reveal the sensitivity and specificity of AI in the DR screening of the community population. Comprehensive AI Retinal Expert [CARE, Shanghai EagleVision Medical Technology Co., Ltd. (Airdoc)] is a model for a single convolutional neural network. Compared with other deep learning systems, it is more efficient and has a lower computational cost. A real-world evidence study showed the practicality of CARE in the 14 most common retinal abnormalities (including referable DR, referable hypertensive retinopathy, glaucomatous optic neuropathy, pathological myopia, retinal vein occlusion, et al.) (16).

However, there is currently no research report on the application of this AI system to screen for DR in Chinese community healthcare centers. Therefore, in this study, we report the sensitivity and specificity of the artificial intelligence in screening for DR in a Chinese community population. The CARE module for DR used in this study was approved by the National Medical Products Administration in China.

## Methods

### Study Design

**Figure 1:**
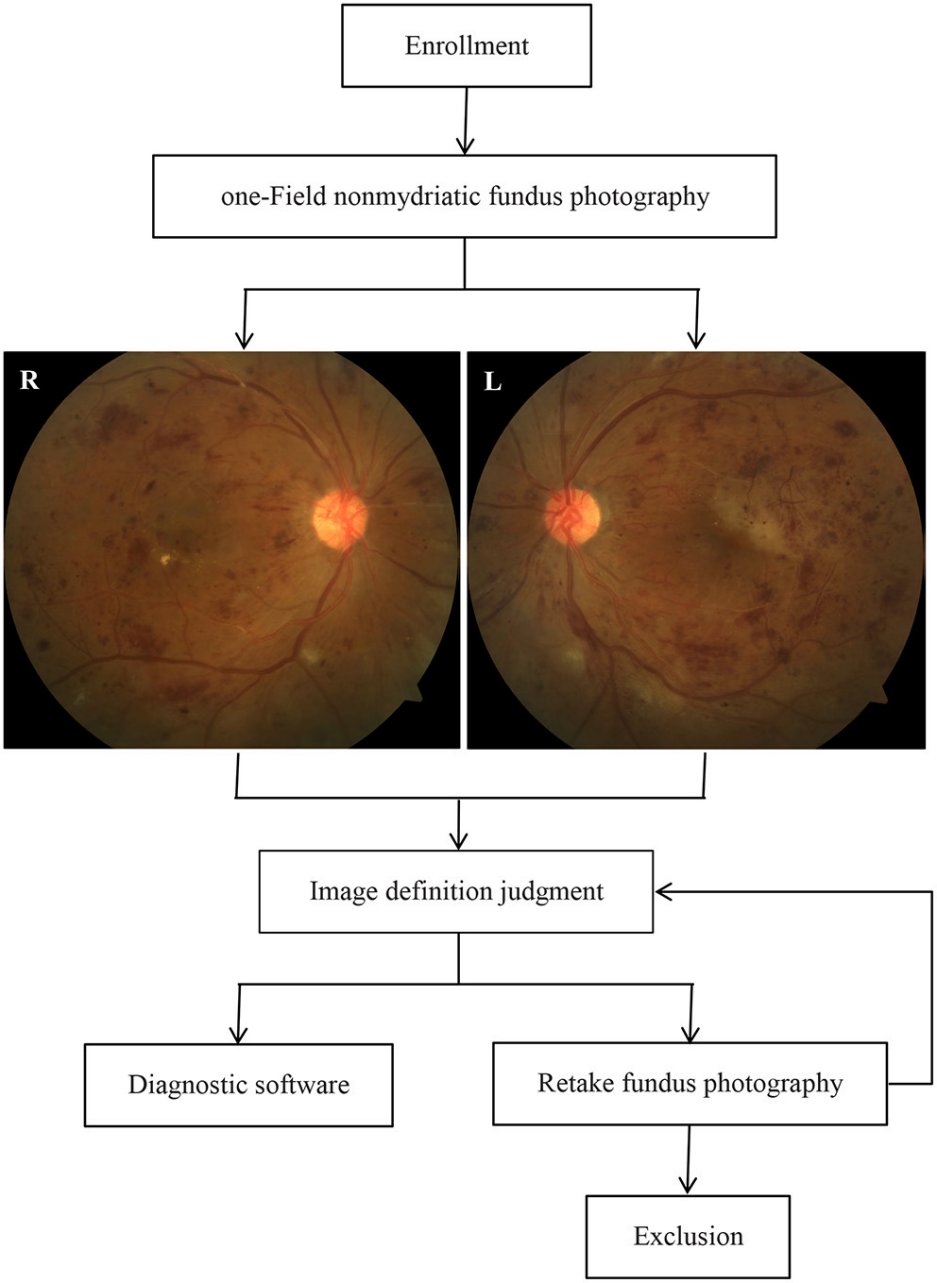
Study procedures in the study of the CARE system.

This study was conducted as a cross-sectional diagnostic study that was registered (ChiCTR2100054663) in the Chinese Clinical Trial Registry between June 2019 and November 2021 in three Chinese community hospitals. The overall study design is shown in Figure 1 and is described herein. The study protocol was approved by the Ethics Committee of Dongguan Tungwah Hospital (2019DHLL046) and was conducted in compliance with the Declaration of Helsinki. All subjects provided informed consent and their fundus images were anonymized.

### Study Subjects

The study used an artificial intelligence system to assess fundus photography of DR in a sample of 443 individuals (848 eyes) from Chinese community healthcare centers with a previous diabetes mellitus (including type 1 and type 2 diabetes mellitus) diagnosis. Subjects were aged 18 years or older. Exclusion criteria included pregnant or lactating women, psychiatric disorders, retinal vascular disease other than DR, history of intraocular treatment (including intraocular injection, retinal laser photocoagulation, and vitrectomy, but excluding uncomplicated cataract surgery), and glaucoma. Those with media opacities resulted in poor-quality images, such as keratitis or severe cataracts.

### Image Acquisition and Reference Standard Grading

One-field color fundus photography (CFP) (macula-centered with a 50° field of vision) was taken for both eyes using a non-mydriatic fundus camera (RetiCam 3100, China) by 3 trained ophthalmologists in darkrooms. Images were collected by a professional technician at each hospital. At least one fundus photograph should be obtained to ensure good image quality. A good quality image needs to meet the following conditions (17) at the same time, otherwise it is defined as a poor quality image: (1) Except for DR-related signs such as fibroproliferative membrane, preretinal hemorrhage, and vitreous hemorrhage, 90% of the blood vessels in the image can be identified; (2) The field of the image is not <50° in both the horizontal and vertical directions. The distance from the fovea to the center of the image is <1 optic disc diameter and more than 2 optic disc diameters from the fovea to the edge of the image; (3) There are no shadows and reflective area that affect image interpretation; (4) The image is not overexposed or underexposed. All images were uploaded to an online artificial intelligence system and submitted to two independent attending ophthalmologists for analysis.

Both ophthalmologists and the AI system graded the images according to the International Clinical Diabetic Retinopathy (ICDR) classification criteria: no DR, mild non-proliferative DR (NPDR), moderate NPDR, severe NPDR, proliferative DR (PDR), and unrecognizable. Concurrently, all images were classified with or without clinically significant macular edema (CSME). The results were compared between ophthalmologists to assess the intergrader agreement. Disagreements between the two ophthalmologists were adjudicated by a third more senior retinal specialist for the final grade. The “ground truth” defined as the gradings made by the two ophthalmologists without disagreement or gradings made by the senior if disagreements exist. The AI performance was then evaluated and compared with the ground truth. For analysis, the final ICDR grades were combined into any DR (including mild NPDR, moderate NPDR, severe NPDR, and PDR), more-than-mild DR (mtmDR, including moderate NPDR, severe NPDR, and PDR) and vision-threatening DR [vtDR, including severe NPDR, PDR, and presence of diabetic macular edema (DME)].

### Outcome Measures

Primary outcome measures included the sensitivity, specificity, positive predictive value (PPV), negative predictive value (NPV), and their 95% confidence intervals (CIs) of the AI system for detecting DR and DME.

The mtmDR, vtDR, and DME results were independently examined. Sensitivity was defined as the accuracy of positive findings per reference standard and specificity as the accuracy of negative findings per reference standard. Sensitivity, specificity, PPV, and NPV were calculated as follows: sensitivity = TP/(TP + FN), specificity = TN/(TN + FP), PPV = TP/(TP + FP), NPV = TN/(TN + FN). Image ability was defined as the percentage of eyes that received a disease detection result from the AI system (positive or negative) among all images determined gradable by the graders.

### Statistical Analysis

The images grading results were collected in Microsoft Excel 2017 files, and statistical analyses were conducted using the IBM SPSS Statistics 19 software (SPSS Inc., Chicago, IL, USA). Values conforming to a normal distribution were expressed as mean ± standard deviation, while values that did not conform to a normal distribution were expressed as median and quartiles (median [interquartile range]). Statistical significance was set at *p* < 0.05.

## Results

In this study, 445 individuals signed informed consent forms and 443 participants (848 eyes) completed the study according to the protocol. Of the participants, 63.88% were male. The demographic characteristics of the subjects are listed in Table 1.

**Table 1:** Demographic characteristics of the subjects.

**Subgroup**	
Age, years	52.09 ± 11.51
**Sex**	
Men (%)	283 (63.88%)
Women (%)	160 (36.12%)
Course of diabetes mellitus, years	5.08 ± 5.01
BMI	24.52 ± 3.62

*BMI, body mass index*.

According to the reference standard per the ICDR grading system, of the 848 eyes, 582 (68.63%) had no DR, 33 (3.89%) had mild NPDR, 121 (14.27%) had moderate NPDR, 68 (8.02%) had severe NPDR, and 44 (5.19%) had PDR. For DME, 56 (6.60%) eyes were diagnosed with CSME by the ophthalmologist. The AI system successfully graded 840, with an image ability of 99.06% (8 eyes were judged as unrecognizable by the AI system due to the presence of signs such as vitreous hemorrhage that reduced the image clarity). Further analyses of the sensitivity, specificity, PPV, and NPV for the AI system to detect any DR, mtmDR, and vtDR are shown in Table 2.

**Table 2:** CARE performance for detecting DR.

	**Any DR (95%CI)**	**mtmDR (95%CI)**	**vtDR (95%CI)**
	**(*N*/Total *N*)**	**(*N*/Total *N*)**	**(*N*/Total *N*)**
Sensitivity	75.19 (69.47–80.17) (200/266)	78.97 (73.06–83.90) (184/233)	33.93 (25.41–43.56) (38/112)
Specificity	93.99 (91.65–95.71) (547/582)	92.52 (90.07–94.41) (569/615)	97.69 (96.25–98.61) (719/736)
PPV	85.11 (79.76–89.28) (200/235)	80.00 (74.12–84.85) (184/230)	69.09 (55.03–80.48) (38/55)
NPV	89.23 (86.44–91.52) (547/613)	92.07 (89.58–94.02) (569/618)	90.67(88.37–92.56) (719/793)

*CARE, Comprehensive Artificial Intelligence Retinal Expert; DR, diabetic retinopathy; mtmDR, more-than-mild diabetic retinopathy; vtDR, vision-threatening diabetic retinopathy; N, number; PPV, positive predictive value; NPV, negative predictive value*.

DME is one of the most frequent vision-threatening treatable complications of DR; the sensitivity, specificity, PPV, and NPV for the AI system to detect DME are shown in Table 3.

**Table 3:** CARE performance for detecting DME.

	**DME (95%CI) (*N*/ Total *N*)**
Sensitivity	47.37 (34.18–60.91) (27/57)
Specificity	93.99 (91.65–95.71) (547/582)
PPV	20.00 (13.80–27.93) (27/135)
NPV	95.95 (94.19–97.20) (710/740)

*CARE, Comprehensive Artificial Intelligence Retinal Expert; DME, diabetic macular edema; N, number; PPV, positive predictive value; NPV, negative predictive value*.

## Discussion

This cross-sectional diagnostic study in China explored the performance of the CARE system, and the results revealed that this AI system was efficient in detecting any DR, mtmDR, and vtDR in diabetic patients through a practical application in community healthcare centers. Using single-field CFP imaging, the AI system demonstrated superior specificity in detecting any DR, mtmDR, and vtDR and showed relatively high sensitivity in detecting any DR and mtmDR. This study also demonstrated that the AI system has good maneuverability and credibility for screening DR in the real world. The artificial intelligence DR screening system helps to provide primary retinal screening for diabetic patients in communities, which could prevent diabetic patients from potential visual threat and help to reduce the DR disease burden. To the best of our knowledge, this is the largest real-world study of community-based artificial intelligence screening for DR in Chinese community healthcare centers.

In this study, the sensitivity and specificity of AI for detecting any DR were 75.19% (95% CI 69.47–80.17) and 93.99% (95% CI 91.65–95.71), respectively. The sensitivity and specificity for detecting mtmDR were 78.97% (95% CI 73.06–83.90) and 92.52% (95% CI 90.07–94.41), respectively. This shows that this AI system has a high application value for community screening of any DR and mtmDR. However, the AI system showed a lower sensitivity of 33.93% when it comes to detecting vtDR. We believe that this system still has certain limitations in identifying severe PDR and DME and speculate that there are several reasons for this result: (1) Li et al. (18) found that vascular damage and new blood vessels in the PDR are more distributed in the nasal field. We speculate that some lesions were missed in the 50-degree fundus photography centered on the macula. (2) Some fundus photographs have poor imaging results, such as ghost images and blurred lesions, especially in cases of leukoplakia, lens opacity, and small pupils, which make it difficult for AI to identify. Concurrently, it is difficult to detect retinal thickening in the macular area in plane images and to make a correct diagnosis (19). (3) The insufficient sample size in this study led to the deviation in the results. Nevertheless, the high recognition rate of AI for mtmDR detection makes it a good practical application value. The application of the AI system helps to enable timely intervention in fundus retinopathy that threatens the vision of diabetic patients and has good practical application significance.

The application of AI screening systems in communities or primary health care institutions often faces difficulties due to the lack of professional image acquisition technicians and lack of cooperation from patients. This study verified the feasibility and accuracy of an AI DR screening system in community healthcare centers. After standardized training, the community health personnel can master the use of the system, and general practitioners can complete the collection and uploading of images without mydriasis or invasive operations. The AI system can rapidly analyze fundus images and generate diagnostic reports and referral recommendations in minutes. Patients who need further examination and treatment will be referred, and other patients will continue to be followed up and observed. This mode of diagnosis and treatment helps reduce time and economic costs for patients and helps reduce the pressure on superior hospitals (20, 21). The convenience and timeliness of image reading by the artificial intelligence system enable patients to have good compliance and participation. This helps prevent patients from delaying medical treatment because of difficulties in seeking medical care, which can lead to severe visual impairment (22, 23). In addition, the system can also be applied to the follow-up management of diabetic patients without DR and with mild NPDR, which helps to improve the medical compliance of patients.

Raju et al. (24) reported a sensitivity of 80.28% and a specificity of 92.29% for automatic diagnosis of DR on the publicly available Kaggle dataset. Our research showed similar results to this study. For detecting mtmDR, an external validation study (16) of the CARE system from 35 medical institutions (including 8 tertiary hospitals, 6 community hospitals and 21 physical examination centers) reported a sensitivity of 93.8% and a specificity of 87.8%. Unlike our study, the data for this study were mainly collected from large medical institutions and the system for detecting community-based mtmDR has not yet been reported. Ipp et al. (25) reported the performance of an artificial intelligence system (the EyeArt Automated DR Detection System, version 2.1.0) for detecting vtDR: the sensitivity was 95.1% and the specificity was 89.0%. However, it is worth noting that the results of this study are not entirely based on community diabetes, and the AI system was evaluated using two-field fundus photography. Compared with the above studies, the sensitivity of our research was lower. However, our research was entirely based on community data, which may better reflect the real situation of artificial intelligence applied to the screening of diabetic retinopathy in the Chinese community. On the other hand, it reminds us that for real-world application scenarios, the CARE system still needs to further improve its algorithm for higher diagnostic accuracy.

The limitation of this study are the limited sample size and single-field color fundus photographs which may lead to a certain missed diagnosis rate. Therefore, further well-designed multicenter studies with large samples and different ethnic backgrounds are warranted to further assess this AI system.

In conclusion, this cross-sectional diagnostic study revealed the reliability of the CARE system for DR screening in community health care centers. The system can effectively improve the screening capability of community healthcare centers for DR and help build an early detection system for DR based on AI systems.

## Data Availability Statement

The raw data supporting the conclusions of this article will be made available by the authors, without undue reservation.

## Ethics Statement

The studies involving human participants were reviewed and approved by Ethics Committee of Dongguan Tungwah Hospital. The patients/participants provided their written informed consent to participate in this study. Written informed consent was obtained from the individual(s) for the publication of any potentially identifiable images or data included in this article.

## Author Contributions

XD contributed to the study design and wrote the manuscript. SD contributed to the study design, data analyses, and manuscript polishing. WZ contributed to the data analyses, and manuscript preparation. CC contributed to the conception of the study and manuscript polishing. HL and JZ assisted in clinical data and images collection. All authors read and approved the final manuscript.

## Funding

This study was supported by the Social Science and Technology Development (Key) Project in Dongguan (202050715046227).

## Conflict of Interest

The authors declare that the research was conducted in the absence of any commercial or financial relationships that could be construed as a potential conflict of interest.

## Publisher's Note

All claims expressed in this article are solely those of the authors and do not necessarily represent those of their affiliated organizations, or those of the publisher, the editors and the reviewers. Any product that may be evaluated in this article, or claim that may be made by its manufacturer, is not guaranteed or endorsed by the publisher.
